# Education in times of restriction: an examination of refugee girls’ and young women’s access to learning during COVID-19 school closures in Pakistan

**DOI:** 10.1007/s10671-023-09353-4

**Published:** 2023-06-16

**Authors:** Katrina Barnes, Amy Ashlee, Aimee Mukankusi, Julia Pacitto, Asma Rabi, Matt Thomas, Noor Ullah, Rozina Zazai, Annette Zhao

**Affiliations:** 1 Jigsaw, London, UK; 2 Refugee Education UK, London, UK; 3 EdTech Hub, London, UK

**Keywords:** COVID-19, Gender, Pakistan, Refugee education, Educational technology, Distance education

## Abstract

This paper examines the extent to which refugee girls and young women were able to access learning during COVID-19 education closures in Pakistan, and the role that EdTech played in their learning access. It is based on findings from a survey with 403 Afghan refugee students, along with in-depth interviews with six young female refugees. The research shows that, while the majority of female refugee students were able to continue accessing education in some form during school closures, learning access was nevertheless limited, and a sizable minority were not engaged in any learning during this time. Teacher and institutional support was either absent or inadequate for many students, and infrastructure and devices that serve to support remote learning were not always reliable or accessible. Although male respondents were less likely than females to engage in independent study during the closures, refugee girls and young women were significantly less likely than their male counterparts to own the devices they needed for learning. The findings demonstrate how targeted investment in specific types of EdTech and teacher professional development, as well as supporting educational institutions in the establishment of remote learning opportunities, could help sustain learning during future periods of educational restriction.

## Introduction

The COVID-19 pandemic caused unprecedented disruption to education around the world. Social distancing measures forced schools and universities to close, affecting 1.6 billion learners globally (UNESCO, 2021). Educational technology (EdTech)^1^ became indispensable, with teachers and educators relying on a range of digital technologies to provide remote learning and ensure educational continuity (Dreesen et al., 2020). While school closure policies were almost ubiquitous, not all learners were affected by these equally, with disadvantaged children and young people disproportionately less able to access technology and education (Avanesian et al., 2021; Di Pietro et al., 2020; Jordan et al., 2021; Reuge et al., 2021).

Refugee girls and young women are situated at the intersection of two significant inequalities that inhibit their likelihood of accessing and progressing in education—being female and being forcibly displaced from their home countries (Canetti, 2020; UNHCR, n.d.). There is widespread concern that refugee girls and young women will experience severe effects on their education as a result of COVID-19 (UNHCR, 2020a). While evidence emerges on the impact of the pandemic more broadly, relatively little is currently known about refugee girls’ and young women’s educational experiences during school closures and their interactions with EdTech.

This paper contributes evidence to this gap through an examination of Afghan refugee girls’ and young women’s experiences of secondary and higher education in Pakistan during the COVID-19 pandemic and the role of technology within this. It is especially important to understand if and how girls were able to continue learning in this context, and what that may mean for related contexts given that, at the time of writing, there are significant challenges and ongoing uncertainty regarding access to secondary and higher education for girls in Afghanistan due to the recent regime change.

The paper addresses three questions:

1. How and to what extent were Afghan refugee girls and young women in Pakistan able to access education and learning at secondary and higher levels during the COVID-19 pandemic?
2. What role did technology play in supporting these girls and young women to access education and learning at this time?
3. What improvements can be made to better support Afghan refugee girls and young women to access education and maintain learning during future crises and periods of educational restriction?

## Research context

Pakistan has a long history of hosting Afghan refugees. Between 1979 and 2001 an estimated 4.4 million Afghan people sought refuge in Pakistan (RAHA, 2016) and, as of December 2020, 1.4 million Afghan people remained in protracted forced displacement (UNHCR, 2022). Afghan refugees are predominantly located in the northwest of Pakistan in Khyber Pakhtunkhwa (UNHCR, 2020b). The regime change in Afghanistan in the summer of 2021 instigated renewed forced displacement, with an estimated 117,550 new Afghan refugees arriving in Pakistan between January 2021 and February 2022 (UNHCR, 2022).

Education for Afghan refugees is situated within what has been described as Pakistan’s “extensive education crisis” (Naviwala, 2016), characterised by high numbers of children out of school. In 2018, just 37% of secondary school-aged children were enrolled in school (World Bank, n.d.). Although Afghan refugees in Pakistan have access, in principle, to the national education system, significant challenges limit their ability to access and thrive in education. While data on Afghan refugees’ enrollment in education are scarce (Hervé, 2018), enrollment rates in education have been particularly low in Pashtun areas on the border of Pakistan and Afghanistan (Jamal, 2014). Available estimates suggest that between 5 and 20% of refugee youth aged 15–24 attend school (Hervé, 2018, p. 5).

The COVID-19 pandemic exacerbated this "education crisis" for refugees and the wider population in Pakistan. From March 2020, education institutions experienced a cumulative 61 weeks of lockdowns (UNESCO, 2021), with closures and reopenings broadly mirroring the trajectory of various waves of the COVID-19 pandemic in Pakistan (Kamran et al., 2022). Normal educational instruction did not formally resume until October 2021.

National and provincial governments in Pakistan rapidly implemented a multimodal approach to promoting educational continuity once schools had closed, including by drawing on radio, SMS, TV and digital learning platforms (Zubairi et al., 2021). Meanwhile, the Higher Education Commission of Pakistan (HEC) instructed universities across the country to provide online teaching for students (Iqbal et al., 2022). Nevertheless, it is predicted that children and young people in Pakistan will experience significant learning losses and high levels of dropout linked to the education closures (Geven and Hasan, 2020).

Existing literature indicates that, within this broader landscape of refugee education in Pakistan, gender dynamics function to impact both girls’ and young women’s access to education and learning, and access to technology that can support their learning. This literature is further explored in the two subsections below.

### Refugee girls’ access to education and technology in Pakistan

Before the pandemic, Pakistan experienced significant gender disparities in educational access (Denham et al., 2020). According to Human Rights Watch’s, 2018, report, 59% of girls in Pakistan are out of school by the time they reach grade 6 (11 years old), versus 49% of boys, and only 13% of girls are still in school by grade 9 (14 years old) (Human Rights Watch, 2018). Furthermore, the World Bank’s gender parity score for enrollment in tertiary education was 0.87 (World Bank, n.d.), suggesting that women and girls were disadvantaged in their access to university.

Gender inequality is also evident in available statistics on Afghan refugees’ education in Pakistan. Across 171 UNHCR-funded schools in refugee villages, only 18% of girls were enrolled in primary and secondary school in 2017 (UNHCR, 2017, p. 14). While data disaggregated by the level of education are not available, the gender disparity in refugees’ access to secondary education has been reported as among the worst in the world (UNHCR cited by Zubairi and Rose, 2016, p. 12). There are also significant gender gaps in literacy rates among Afghan refugees. Research with Afghan returnees from Pakistan to a bordering province in Afghanistan found that only 7% of women over the age of 15 were literate as compared to 38% of men (Samuel Hall, 2017, cited in Hervé, 2018, p. 6).

A broader landscape of gender discrimination across Pakistan underpins refugee girls’ access to education (Human Rights Watch, 2018), including child marriage and teenage pregnancy (Zubairi and Rose, 2016), restrictions in girls’ freedom of movement (UNHCR, 2017) and limiting gender roles (Asghar et al., 2018; Jamal, 2014). This gender discrimination intersects with other barriers to refugee girls’ education—including poverty, risks on journeys to and from school, limited numbers of places for Afghan refugees in government schools, the high cost of education and a lack of female teachers (Asghar et al., 2018; Denham et al., 2020; Human Rights Watch, 2018; Jamal, 2014; UNHCR, 2020b)—creating what has been described as an “upward bottleneck” for girls as they progress through the educational stages (Human Rights Watch, 2018, p.13).

Girls’ access to technology is also affected by these socio-cultural gender dynamics. The literature suggests that, in societies with restrictive gender norms such as Pakistan and Afghanistan, technology within households is often reserved for and controlled by men and boys (Malala Fund, 2020). In addition to gender norms, practical constraints—whereby access to technology is absent or extremely limited—are reported as a significant barrier hindering girls’ ability to access and benefit from technology (Crompton et al., 2021). Indeed, despite significant improvements in women and girls’ access to technology in Pakistan since 2017, it is reported that the gender digital divide is still prominent (Shanahan, 2021). A recent report from GSMA (ibid., p. 3) found that only 50% of women own a mobile phone in Pakistan compared to 81% of men, and that women are 49% less likely than men to use mobile internet—reportedly some of the highest levels in the world. Shanahan (2021) finds that family disapproval of women and girls’ access to technology is a significant driver of the gender digital divide in Pakistan.

### Refugee girls’ access to learning in Pakistan during the COVID-19 pandemic

While limited, some emerging evidence sheds light on the gendered nature of learning during COVID-19 lockdowns in Pakistan. One the one hand, a survey carried out by Malala Fund in the summer of 2020 with 1598 households across all four provinces (including Khyber Pakhtunkhwa where the majority of Afghan refugees live) found that girls were more likely than their male counterparts to be required to undertake chores and caregiving at the expense of their education and remote learning (Denham et al., 2020). A randomised control trial (RCT) conducted in Punjab between November 2020 and August 2021 also found that, while the majority of the 246 parents of grade 8 children surveyed did not report restricting girls’ access to EdTech, over a fifth (22%) did report prioritising boys’ access to technology for education, and nearly a third (29%) reported restricting girls’ access to devices needed for completing online education (Adil et al., 2021, p. 35). The justifications reported for these restrictions were cultural, religious and financial (ibid). However, findings from a separate survey with 1528 households with children enrolled in a private schools network run by The Citizens Foundation (TCF) in 2020 and 2021 suggest that girls consistently spent more time studying, and had made better learning progress than boys (Crawfurd et al., 2021).

Overall, the evidence base is currently unclear, and the lack of data on EdTech and access to education in Pakistan disaggregated by both genders and refugee status demonstrates the need for further robust research to fully understand gendered experiences of refugee girls and young women and their engagement with EdTech during COVID-19. This research contributes to addressing the gap in evidence.

## Research methods

To examine how refugee girls and young women made use of EdTech to sustain learning during the school closures associated with COVID-19 in Pakistan, the study combined a survey with 403 refugee students and semi-structured interviews with six young refugee women. All data were collected by youth researchers who are Pakistan-based Afghan refugees. Refugee researchers were trained on the importance of confidentiality, anonymity, informed consent and data protection, and these principles were adhered to at all stages of the research process. Oral consent scripts, which included information about the study and the terms of the research, were read to prospective participants, and informed consent was obtained in advance of any data collection that took place.

### Survey data collection and analysis

The quantitative data used in this analysis were extracted from survey data within a longitudinal panel study tracking the education journeys of secondary and higher education refugees students in Pakistan.^2^ Participants were surveyed at three data points. While data point 1 (DP1) took place prior to COVID-19, data points 2 (DP2) and 3 (DP3) took place in the midst of the pandemic, during which time there were extended school and university closures. Therefore, only DP2 and DP3 data are analyzed in this study. DP2 took place between October and December 2020 and DP3 between June and August 2021.

Both DP2 and DP3 surveys posed questions relating to students’ education experiences, work experiences, perceptions of educational impacts, future plans and the impact of COVID-19 on their education. Data relating to learning and technology access during closures was extracted from those broader datasets for the purpose of this analysis. The same participants were surveyed at both data points, although there was a degree of attrition.^3^ A selection of DP2 survey questions was repeated at DP3 to allow for insights into changes in opinion during the intervening months, while new questions were also asked at DP3 in light of the changing context.

All students surveyed were refugees living in Pakistan or Afghanistan^4^ at the time of school and university closures and were either in class 12 of secondary school or enrolled in higher education during this period. The final sample size was 403 students at DP2 and 313 students at DP3, of which 16% and 15%, respectively, were females (see Table 1).

**Table 1:** DP2 and DP3 sample composition, by gender

	DP2	DP3
Male (*n*, % of total)	337 (84%)	267 (85%)
Female (*n*, % of total)	66 (16%)	46 (15%)
Total	403 (100%)	313 (100%)

Quantitative data were analysed using R software, which was used to generate descriptive statistics that were disaggregated by gender and education level. Chi-square tests (Lewis-Beck et al., 2004) were also generated to identify the statistical significance of gender differences, and regressions explored relationships between variables. Quantitative data analysis was conducted by the UK-based members of the research team and was then sense-checked by Pakistan-based refugee researchers who contributed contextual detail related to the findings.

### Interview data collection and analysis

Remote semi-structured interviews (Denscombe, 2017) were conducted by the Pakistan-based Afghan refugee researchers in March 2022 to further explore some of the key themes emerging from the survey data. Interviews consisted of questions on participants’ education experiences during COVID-19 closure, and participants’ use of technology to aid their learning during that time. Six female interviewees were identified according to the following inclusion criteria: female; Afghan; refugee; studying at a Pakistan-based institution during closures and either in grade 10 or above of secondary school or enrolled in higher education during closures. Issues around the recruitment of interviewees are further explored in the Limitations section, below.

All qualitative transcripts were translated and coded manually using an inductive approach by a team of both the UK-based researchers and Pakistan-based refugee researchers. These codes were then combined and grouped for thematic analysis (Denscombe, 2017). Once both analyses were complete, key overarching themes from both the quantitative and qualitative findings were compared and contrasted to allow for a more in-depth picture of the key issues posed by the research questions and to enhance the validity of emerging conclusions. The refugee researchers involved in the qualitative analysis were able to add a level of contextual detail and insight that allowed for a nuanced interpretation of the qualitative data.

### Limitations of the research

As would be expected with a study of this nature, there are a range of methodological limitations to note. Firstly, there is a disconnect between the survey sample focus on secondary school students and the interview sample focus on higher education students, with five out of the six interviewees being enrolled at university, and only one at secondary school. This is because female secondary school students were largely unavailable or unwilling to participate in interviews. Researchers, therefore, had to adopt a convenience sampling approach based on the availability and willingness of students. As a result, it has not been possible to make any comparison between different education levels within the analysis.

There was also an uneven gender split within the survey sample, with a smaller number of female respondents than initially hoped. This may be attributable to cultural expectations encouraging women not to voice their opinions to the same degree as men and in some cases not to speak to strangers at all. This was mitigated to some extent by the use of inferential statistics within the analysis, for which sample numbers are taken into account when determining effect size or significance of findings.

Finally, the nature of the insights provided by quantitative and qualitative data is affected by the time period within which they were collected. Quantitative data at both DP2 and DP3 were collected during the period when schools and universities were mainly closed. Qualitative data, however, were collected once education institutions had officially reopened, and interviewees were asked to reflect back on their previous experiences. The implications of this were considered throughout the analysis.

## Findings

The study findings are presented according to the main themes emerging from the analysis. These are learning experiences; learning resources used by female students; barriers to learning for female students and ways to improve learning access. Interview participants are referred to as “P1,” “P2,” etc. throughout. It is important to note that the data have been collected from girls who are enrolled in the final year of secondary education or in higher education as refugees in Pakistan. The findings should not be misunderstood as being indicative of the experiences of refugee girls in Pakistan as a whole, but rather give insight into the educational experiences of the minority of Afghan girls who had managed to progress to the end of secondary and into higher education.

### Learning experiences during closures

The majority of female students reported that they were still engaged in some form of learning (56%) during school closures. However, only 13% of female students engaged in formal study through their educational institution, with 44% engaging in self-directed study (i.e., not guided by school/university). Only one survey respondent reported engaging in both formal and self-directed studies.

Conversely, qualitative interviewees all reported that they were engaged in some form of formal learning, attending online classes (P1, P3, P5 and P6) and/or watching pre-recorded lectures (P2, P3 and P4). This disconnect between qualitative and quantitative findings may be due to the fact that, while the majority of the survey respondents were secondary school students, five out of six qualitative interviewees were university students, and therefore likely benefited from the HEC policy mandating Pakistani universities to provide online teaching for students.

All six interviewees also reported that they had engaged in self-directed study during closures; however, two students (P3 and P6) reported only beginning self-directed study in the second semester of closures due to a lack of motivation earlier on. Notably, reasons that interviewees gave for studying independently were related to the absence or poor quality of formal educational offerings during the closures, including: a lack of online teaching available from the participant’s institution (P4); the low quality of online lessons that were available (P1); and inadequate teaching (P1 and P4).

Among survey respondents, a large majority (84% of those engaged in learning) reported only learning a small amount during school closures. This is largely consistent with qualitative findings: All interviewees reported that they had managed to learn to some extent. However, while one interviewee (P5) reported that the extent of her learning had been more or less the same as prior to COVID-19, she acknowledged that her situation was rare. Indeed, five of the six interviewees remarked that they had learned less during closures than they would have in normal times:

“[The pandemic] badly affected our learning (...) Now we are supposed to do research work, and we don’t have any idea how to do it.” (P6)

Female students were generally more likely to engage in self-directed study during the COVID-19 closures (44%) than males (21%). A Chi-square test conducted on this data indicated that this is a highly significant gender difference statistically and is especially noteworthy given how few students of both genders were engaging in formal education (*Χ*^2^ (1, 389) = 12.389, *p* < 0.001, see Table 2).

**Table 2:** Activities undertaken during closures (select all that apply, *n* = 389, total excluding 14 NAs for this question)

Activity	Females (*n* = 54)	Males (*n* = 335)
Self-directed study	24 (44%)	71 (21%)
Formal education	7 (13%)	32 (10%)
Unpaid work	18 (33%)	40 (12%)
Paid work	2 (4%)	90 (27%)
Nothing	15 (28%)	124 (37%)

Percentages represent students who chose the respective option within each gender group, not across the whole sample; options are not mutually exclusive apart from the “nothing” option, i.e. students could choose multiple options from the first four options, or the last option

As Table 2 indicates, female respondents were significantly more likely to be undertaking unpaid work during the pandemic compared to males, which may have impacted the time that they were able to spend on learning. This may be due to cultural expectations placed on women relating to domestic responsibilities (Price, 2020). Conversely, a much higher proportion of male respondents reported that they were engaged in paid work than females.

### Learning resources used by female students during closures

DP2 survey data suggest that books were the most commonly used learning resource, with 73% of female respondents engaged in learning^5^ choosing this option when asked which study resources they used to sustain their learning while unable to access school. Books were also identified in the qualitative data as important learning resources, though to a lesser extent. Three interviewees (P1, P3 and P5) reported using books for self-study, although P3 and P4 indicated that books were less commonly used than internet resources among their peers. P4 explained that, for her, using books was not easy without teacher guidance:

“Studying school books without teachers’ help or guidance is really hard (...) a teacher is very important to make you understand those books.” (P4)

The finding that books were the most commonly used learning resource could be due to the fact that secondary students, who made up the majority of the survey sample, may be more likely to have limited device and internet access than university students. Higher education students, conversely, are more likely to come from families who are able to afford internet access.

The survey also demonstrates the importance of access to the internet as a resource that supports learning. DP2 data showed that the internet was the second most commonly used resource, with 43% of female respondents reporting that they used online resources to study during closures. In addition, 33% of female respondents reported using the internet to seek support with their learning more generally. The importance of the internet as a resource is also evidenced in the qualitative data; all six interviewees stated that they had used it to study remotely. Of these, four interviewees (P1, P2, P3 and P4) noted that the internet was the most popular resource among their peers, and two interviewees (P4 and P5) noted that the internet was an essential resource upon which their education was completely dependent during closures. Interviewees also cited a variety of platforms and websites that had proven crucial to their learning, including Google for general information (P2 and P4) and YouTube for understanding key topics (P4). Other platforms referenced included WhatsApp, which was used to communicate with teachers and form study groups with peers (P5), and live classes were conducted using Zoom (P1 and P2) and Microsoft Teams (P5).

A survey question asked respondents engaged in learning which devices they considered most important for continuing to learn during the closures: Over half of female students reported that a feature phone (30%) or basic phone (27%) had been the most important device that helped them continue to learn during closures. Twenty-three percent of female students reported that a smartphone was the most important device for them, while only 3% stated that a laptop had been the most important device for them. All six interviewees owned at least a mobile phone, while three interviewees also owned a laptop (P4, P5 and P6). These devices were used to access the internet to study remotely (all interviewees), attend online classes (P1, P3, P5 and P6) and watch pre-recorded lectures (P2, P3 and P4). P3, P4 and P6 also reported using different devices for different purposes within their learning. For example, P4 and P6 reported using their laptops for completing assignments, while both they and P3 used their phones for attending live classes. Notably, almost no girls reported using radio or TV as a learning resource during the school closures (0% and 3%, respectively).

### Barriers to learning for female students during closures

Quantitative and qualitative data show that female students faced a number of learning barriers during COVID-19 closures. These were associated with: financial constraints; lack of device access; lack of internet access and electricity supply issues; limited or absent institutional support; and inadequate teaching quality.

#### Financial constraints

Financial constraints were a significant issue for female students; 54% of female DP3 respondents reported this as their most significant challenge to completing school or university, making this the most commonly selected significant challenge. With DP3 undertaken during the pandemic, 74% of female respondents reported that they associated this challenge with COVID-19 to some extent. Similarly, two interviewees described how financial challenges made it difficult for them to learn as effectively as before the closures, with the pandemic causing either family members (P4) or themselves (P1) to lose their jobs and therefore their ability to pay for their education. As reported by P4:

“During COVID-19 my father’s job was affected and it was really hard for the family to go on as in pre-COVID times so obviously it affected us psychologically somehow.” (P4)

Furthermore, financial constraints were associated with issues of resource access. Three interviewees (P1, P4 and P6) noted that financial challenges were the direct cause of challenges such as internet and device access (P1, P4 and P6), which are explored in the subsequent subsections. It is noteworthy that male respondents were more likely to report financial issues as their main challenge (61%) than females (54%). This could be explained by the fact that males may have, or perceive themselves as having, greater financial responsibility for their families than their female counterparts.

#### Lack of device access and ownership

Male students across the whole sample were more likely to report device access as a key barrier they faced when attempting to learn using technology (41%, compared with 27% of female DP2 survey respondents). However, among students who were actually engaged in learning during the closures, 37% of women and 28% of men identified device access as a key challenge when trying to learn using technology. Only 26% of female students engaged in learning reported that a smartphone (23%) or laptop (3%) was the most important device they used to sustain their learning during the closures, compared with 51% of male students (of which 25% selected laptop). This is indicative of a relative lack of access to higher-tech devices among female respondents engaged in learning.

Respondents engaged in learning were also asked whether they owned the device they considered most important for aiding their studies: Only 36% of females reported that they did, compared to 74% of males. A Chi-square test indicated a highly significant statistical difference between male and female ownership of devices for learning (*Χ*^2^ (1, 103) = 10.623, *p* = 0.001). Significantly, this lack of device access also impacted students’ access to learning materials; a logistic regression revealed that both male and female students who own devices were less likely to report challenges in accessing learning materials (*p* = 0.03), while gender itself was not statistically significant (*p* = 0.1). Table 3 illustrates that the odds of students reporting access issues related to learning materials decreased by 68% for students who owned devices compared to those who did not.

**Table 3:** Logistic regression model for access to learning materials: gender and tech ownership

	Odds ratio	Estimate	Std. error	*z* value	*p* value
(Intercept)	0.26	− 1.34	0.56	− 2.38	0.02*
Gender: male	2.94	1.08	0.66	1.64	0.10
Tech ownership: owner	0.32	− 1.15	0.52	− 2.22	0.03*

* Indicates *p* value < 0.05

In parallel, two interviewees (P1 and P4) noted that access to devices was a key barrier to learning for them and their peers:

“I know a lot of my classmates did not own a phone and it was very difficult for them to learn. They were turning to their family members to borrow their phones and study online.” (P1)

Both reported that their mobile devices were unreliable, rendering them unsuitable for study, and both interviewees also noted that relying only on mobile devices impeded their learning as they were unable to use them to write assignments effectively. The fact that female students tended to be less likely to own their own devices, and were less likely to use higher-tech devices like laptops, may have limited their access to high-quality learning materials during the school closures.

#### Unaffordable and unstable internet access and electricity supply

Survey data indicate that a sizable minority of female students encountered barriers to learning related to internet access. This was reported as a challenge by 33% of female DP2 survey respondents when asked about issues they had faced when attempting to learn using technology. Accessing the internet was also problematic for three of the interviewees (P1, P2 and P6) due to the high cost of good internet packages (P1 and P6) and the unreliability of access in their location (P1, P2 and P6). This latter issue was so problematic that it prompted these three students to frequently change their locations to find better connectivity, which, in turn, further disrupted their studies:

“It wasn't easy because I was living in a rural area and the internet was even worse there than in the urban areas so I had to move to the city to attend my classes but I suffered a lot.” (P2)

When interviewees were able to access the internet, it was often slow and of poor quality (P1, P2, P3, P4 and P6), especially in rural areas (P2). This created other issues, such as being unable to: download pre-recorded lectures and other resources provided by students’ institutions (P4 and P6); attend live classes (P2); deliver graded online presentations (P1) and upload exam papers (P1).

In addition, it appears that some female refugee students stuck in Afghanistan were less able to access learning during closures due to cultural restrictions preventing women from traveling alone. P6, who attempted to continue her studies from Afghanistan during closures, explained:

“Females face more internet issues compared to males because in Afghanistan females can’t go to a net cafe or anywhere to access the internet but males don’t have that problem. They could even go to one spot where they would do group study or take exams.” (P6)

A smaller but still notable number of respondents identified issues with electricity supply as a barrier to learning, with 17% of female DP2 survey respondents and all six interviewees citing this as a major challenge. Electricity outages and load shedding resulted in students missing classes (P1 and P3) and having their classes disrupted (P5). Other electricity-related issues reported were being unable to charge devices (P2, P3 and P6) and being unable to upload completed examinations (P1 and P3). Two participants reported that this issue was especially severe in Afghanistan, where they were stuck during closures due to border restrictions.

#### Limited or absent institutional support

There is a mixed picture from the data in relation to the level of support offered to students by their education institutions during closures. At DP2, 67% of female survey respondents reported that they had received no support from their institution. However, this was not experienced universally; 17% of female DP2 survey respondents reported that they had been supported through access to a full online learning environment. In the qualitative data, only P5 reported that her university had provided a full learning environment. However, other interviewees did reference accessing some online learning support, including live lessons (P1, P3, P5 and P6) pre-recorded lectures (P2, P3 and P4) and recommendations for resources to access independently (P1 and P4).

Nevertheless, five of the six interviewees agreed that their institutions had been unprepared for online learning, which led to a series of shortcomings when it came to student support. Both P1 and P6 reported that there had been significant delays to live classes beginning, while P4 reported that her secondary school had not been able to offer any live classes at all. P6 noted that there appeared to be variation in how quickly universities had responded to closures; some of her friends had begun online classes immediately, while others’ institutions only started offering these after several months.

Despite reports that support was not reliably available from institutions, there was some indication that support was often not requested either. Only 7% of female DP2 survey respondents reported seeking help from their teachers. Rather than appealing to their institutions, female students were more likely to seek support from their peers, with 30% of female DP2 survey respondents seeking help from friends. Similarly, interviewees frequently mentioned their peers as a key source of support to their learning. This peer-to-peer support was often facilitated by technology and included forming WhatsApp study groups (P5) and working together on group projects and presentations (P5 and P6). Peers also supported one another by sharing notes (P1 and P2) and recording lectures for each other (P2).

#### Inadequate teaching quality

At DP2, the most commonly selected change that would have the biggest positive impact on girls’ ability to do well in their education was “financial support” (27%), but by DP3, this had changed to “better teaching” (26%). This finding indicates that some respondents became increasingly unsatisfied with the teaching, or lack thereof, during the school closures. Similarly, interviewees placed significant value on receiving direct teacher support and expressed how self-study had been less effective than normal teacher-led classes, noting the importance of teacher input in accessing quality learning, especially in technical subjects (P1, P4 and P6).

Despite the importance placed on teacher input, the qualitative data suggest that female students’ learning access suffered due to teachers’ inability to adapt to remote learning. Teachers reportedly lacked familiarity and competence with the technology required to deliver effective online classes (P1, P3, P4 and P6). According to P1 and P3, a lack of teacher competence led to low-quality online classes, an issue which P1 noted was so common in Pakistan during closures that it was frequently trending on Twitter during that time.^6^ This may be due to the fact that, given the sudden arrival of the pandemic, there had not been time to provide the appropriate training in advance (Noor et al., 2020). Two interviewees (P4 and P6), however, reported that their institutions had provided training to teachers on how to deliver remote classes, and P4’s teachers were also provided with devices and cameras with which to record lectures.

Four interviewees (P1, P2, P3 and P6) felt that teachers were also unsupportive of students and unreceptive to their needs, while P5 cited reports of unsupportive teachers from her family members. Both P1 and P6 noted that teachers were unsympathetic to students’ internet issues, while P2 added that her teachers were not understanding about why she had missed online classes due to travel issues in Afghanistan. In addition, teachers were often unwilling to share resources with students (P2), give feedback on online examinations (P6) and give clarifications if students had questions (P2). P1 and P3 both added that it was very difficult to communicate with teachers at all:

“If I had any questions I would email my teacher and she would reply after weeks.” (P3)

Relatedly, two interviewees (P1 and P6) reported that a lack of teacher professionalism had had a marked negative impact on their learning during the pandemic. This included teachers skipping online classes (P1 and P6), not following the curriculum (P1), focusing on getting to the end of the course rather than paying attention to the quality of their teaching (P6) and not checking students’ work for plagiarism (P1). In addition, two other interviewees (P3 and P4) indicated that the lack of teacher monitoring led students to become disengaged and unmotivated during the school closures. Though the pandemic may have simply exacerbated pre-existing tendencies in this respect, these shortcomings could also be explained by suggestions that teachers in Pakistan felt the ill effects of the pandemic on their own lives and mental wellbeing (Noor et al., 2020).

In contrast, one interviewee (P5) explained how her teachers had successfully provided support for students’ learning during this period. They had communicated with students via WhatsApp groups, recommending resources and generally being available to address student concerns. P5, who received access to sustained remote support and a full online learning environment during the closures, was also the only participant who expressed that she was able to learn as much during the closures as she did during normal times. This points to the importance of such mechanisms in sustaining learning during times of educational disruption.

### Ways to improve learning access in the future

In light of their recent experiences of education during COVID-19 closures and also the developing situation in Afghanistan and Pakistan, survey respondents and interviewees provided suggestions for positive changes that could be made to maintain refugee girls’ access to learning in times of crisis (RQ3). The three most common suggestions emanating from the data were: (i) provision of financial support; (ii) improvements to teaching; and (iii) increased access to resources for learning.

While financial support was the most commonly chosen positive change at DP2 (27% of respondents), better teaching rose to the top by DP3 (26%). Related to the findings around inadequate teaching during the pandemic, three interviewees (P1, P3 and P5) identified teacher training as a key area for improvement that would lead to increased learning during closures. This included general preparation for teaching during challenging times (P3 and P5) and specific training on how to use technology as part of their teaching (P1).

The need for increased access to resources for learning was the second most commonly identified change that would positively impact female students’ educational success at both DP2 and DP3 (22% and 20% of female respondents, respectively). Interviewees considered the two main resources to be access to the internet and access to devices for learning. Three interviewees (P1, P4 and P6) cited providing reliable internet access as a key resource that should be made more accessible, with P6 advocating that this could be achieved through government subsidised provision of high-quality, low-cost internet packages to students. Similarly, four of the six interviewees (P1, P3, P4 and P5) identified increasing students’ access to appropriate devices as an important way of sustaining learning. P1 and P4 observed that having some kind of device was crucial to being able to study, explaining that those students who could not access one were effectively prevented from continuing to learn. Three (P1, P3 and P5) specified that it was especially valuable for students to have access to laptops. In addition, P1 and P4 felt that devices needed to be of good quality with a reasonable battery life in order to be effective as an educational tool.

Other recommendations for education institutions included: increasing forward planning for similar situations (P4); increasing pastoral support for students (P3); and providing more resources such as lesson recordings (P6). Recommendations related to the role of government provision included: increasing reliability of electricity supplies (P5 and P6); only closing schools as a last resort (P4); and reducing border restrictions for students trying to travel back to Pakistan from Afghanistan to enable their on-going education (P2).

## Discussion

The data presented in the section above paint a nuanced picture of Afghan girls’ access to learning in Pakistan during COVID-19. The findings suggesting that girls were significantly more likely than boys to be engaged in self-directed study during school closures at first sight appear to contradict commonly held assumptions about refugee girls’ relative lack of access to education. However, they do correspond with other emerging data from the pandemic that shows that girls in Pakistan were more likely to engage in self-study than boys (Crawfurd et al., 2021). According to the above findings, although girls were more likely to engage in unpaid work, boys were more likely to have undertaken paid work during this period, which likely necessitated them to be outside the home and thus away from opportunities for self-study. Relatedly, the data also revealed that boys were also significantly more likely to report financial challenges than girls. This is potentially reflective of the relatively greater level of financial responsibility placed on boys and young men, which may have been particularly acute during COVID-19 and the period of significant economic hardship that it triggered. Refugees have no legal right to work in Pakistan and thus work primarily in the informal sector (Mielke et al., 2021), which the ILO reports were “significantly impacted’ by the COVID-19 pandemic with informal workers suffering massive income losses (ILO, 2020).” Refugee boys and young men, as a result of this economic insecurity, may, therefore, have faced additional responsibilities and pressure to financially support their families during this period which prevented them from studying.

Boys were, however, also more likely to report doing “nothing” during the school closures, which is suggestive of other complex gendered dynamics at play that made boys relatively less likely to engage in learning during the COVID-19 school closures. Further qualitative, gender-sensitive research with both girls and boys, both refugees and the broader community, is needed to explore the reasons behind this.

Conversely, and in line with other emerging research on the girls’ learning during the pandemic in Pakistan (Denham et al., 2020), girls were more likely to be engaged in unpaid work during the pandemic than boys. Evidence of a persistent digital gender divide among Afghan girls and boys also emerged from the data, which showed a highly significant statistical difference between male and female ownership of devices for learning, with this lack of device ownership also impacting students’ access to learning materials. This corresponds with broader data on male and female device ownership in Pakistan, which is suggestive of a significant digital divide (Shanahan, 2021). Challenges related to device access could be explained by the fact that female students may be unable to afford the high price of reliable mobile devices, which the literature suggests is “a significant affordability challenge” for individuals with low incomes in Pakistan who do not have access to finance (GSMA, 2019). These findings may also be partly explained by a cultural prioritisation of male education over female (Sinclair, 2007) and cultural attitudes restricting women and girls’ access to technology (Shanahan, 2021).

Finally, the finding that almost no girls reported using radio or TV as a learning resource during the school closures (0% and 3%, respectively)^7^ is particularly notable relative to other research emerging from Pakistan during COVID-19 which suggests that over a fifth of students from a sample of Pakistani private schools accessed the government’s teleschool programme during the pandemic (Crawfurd et al., 2021). This is potentially indicative of a gap in access to educational support specific to Afghan refugees. While this could partly be explained by lower rates of TV ownership within refugee households, it may also relate to the fact that the majority (93%) of secondary school students interviewed at the outset of the study (during DP1) were enrolled at Afghan private schools, which follow the Afghan curriculum.^8^ Because of this, the remote learning opportunities provided by national and provincial governments in Pakistan during the closures were unlikely to have been accessible or relevant for many Afghan students, leaving them without access to this form of learning support and further compounding the challenges that refugee girls and young women faced in accessing education and maintaining learning during the pandemic.

## Conclusion and recommendations

While over half of refugee girls and young women who had been engaged in formal education before the pandemic were able to continue learning in some form during closures in Pakistan, learning access was still limited for the majority, and only a small minority were engaged in formal education during this time. Teacher and institutional support was either absent or inadequate for many refugee students, and infrastructure and devices that serve to support remote learning were not always reliable or accessible to refugees. Students’ gender also affected learning access in different ways. Although male respondents were overall less likely than females to engage in self-directed study during the closures, refugee girls and young women were significantly less likely than their male counterparts to own the devices they needed for learning. In light of these findings, several key recommendations emerge from this study that may be applicable to future situations of educational disruption for Afghan refugees in Pakistan. These recommendations are context-specific, but may also provide helpful learning for other contexts.

First, it is recommended that the appropriate stakeholders either provide increased financial support for refugee students or work to make internet access and device access more affordable and reliable, with a particular focus on device access for refugee girls and young women. This does not contradict the recommendation of the Smart Buys paper that identifies widespread device distribution—focusing on hardware alone—as being a “bad buy” or poor value for money (GEEAP, 2020, p. 19). Rather, it provides nuance to the recommendation and identifies how, in certain contexts where access to conventional schooling is not possible, and at higher levels of education (secondary and higher), access to appropriate devices and associated enablers is valuable for sustaining learning. This builds on other recent findings that show how targeted provision of devices has had benefits for learning during school closures (GPE Secretariat, 2022; World Bank, n.d.).

Second, findings relating to both RQ1 and RQ2 suggest that many Afghan students did not receive any support for learning from their institutions during closures. This may be attributed to the unexpected nature of the pandemic and a lack of existing digital infrastructure that could be harnessed to support remote learning. It is unlikely that those without existing online environments for their students would have had time to begin provision from the start of closures, and although online classes were eventually offered in some cases, this was not the case everywhere. This absence of institutional support poses particular challenges for refugee learners in Pakistan, most of whom were also unable to benefit from the remote learning provision for secondary school students set up by national and provincial governments. While self-directed study emerged as a relatively successful learning option for certain motivated students, the data indicate that students would have benefited from a much greater level of institutional and teacher support than was often provided. It is, therefore, recommended, to the extent possible, that institutions serving refugees in Pakistan are supported to provide full access to online learning environments, resources and support staff in contexts of educational restriction. In circumstances where this is not feasible, stakeholders working in refugee education should consider harnessing alternative, lower-tech remote learning tools targeted specifically toward refugee students and tailored to their specific learning needs in terms of curriculum and language of instruction.

Third, it is also recommended that education institutions serving refugees in Pakistan prioritise teacher professional development to increase their technological knowledge and skills, but also their awareness of and willingness to respond to student needs, both of which have been revealed to be vital in closure situations. In addition, while it is unclear whether teachers exhibited low levels of professionalism due to believing themselves to be unaccountable or due to a lack of support from their institutions, measures should also be taken to increase both teacher accountability and support for teachers at an institutional level.

Finally, it is hoped that an increased awareness of the gender-specific barriers preventing students from accessing education in times of restriction may help stakeholders to increase their understanding of different students’ issues and subsequently to adapt their support accordingly in order to sustain learning more effectively in the future.

## Footnotes

1 This paper uses Hennessy et al.’s (2021) definition of EdTech, that is “technologies—including hardware, software, and digital content—that are either designed or appropriated for educational purposes” (Hennessy et al., 2021, p. 8).

2 This is an ongoing multi-year research study on refugee education called ‘Voices of Refugee Youth: impact of post-primary refugee education', funded by Dubai Cares.

3 A small number of students (*n* = 28) were unable to be traced during the original DP2 collection, but later reappeared at DP3. This group was asked the DP2 questions at that point, and these data were then added to the pre-existing DP2 data. It should, therefore, be noted that, while the majority of DP2 data was collected at the same time, a small amount was collected approximately 6 months after the rest.

4 While all students surveyed at DP1 were at that time physically present and enrolled at school or university in Pakistan, a small percentage of students (5%) had traveled back to Afghanistan and were residing there during the school closures (DP2 and DP3).

5 Because of the large proportion of students who were not engaged in any learning during the closures, the sample sizes for female students (*n* = 30) and male students (*n* = 97) engaged in learning were relatively small.

6 The hashtag #ShameOnHEC (Pakistan’s Higher Education Committee) was trending during this period: https://twitter.com/hashtag/ShameonHEC?src=hashtag_click.

7 Figures were similarly low for Afghan boys (1% and 0%, respectively).

8 Across the refugee population more broadly, almost half of Afghan secondary students in Pakistan are enrolled at Afghan private schools (UNHCR, 2017, p. 30).
